# Impact of Human Milk Oligosaccharides and Probiotics on Gut Microbiome and Mood in Autism: A Case Report

**DOI:** 10.3390/microorganisms12081625

**Published:** 2024-08-09

**Authors:** Luana Aldegheri, Feras Kharrat, Andrea Conti, Fabio Monica, Francesca Busa, Giuseppina Campisciano, Nunzia Zanotta, Carolina Cason, Manola Comar

**Affiliations:** 1 Institute for Maternal and Child Health—IRCCS Burlo Garofolo, 65/1 Via dell’Istria, 34137 Trieste, Italy; luana.aldegheri@burlo.trieste.it (L.A.); feras.kharrat@burlo.trieste.it (F.K.); andrea.conti@burlo.trieste.it (A.C.); giuseppina.campisciano@burlo.trieste.it (G.C.); nunzia.zanotta@burlo.trieste.it (N.Z.); carolina.cason@burlo.trieste.it (C.C.); 2 Department of Gastroenterology and Endoscopy, Trieste University Hospital, Strada di Fiume 447, 34149 Trieste, Italy; fabio.monica@asugi.sanita.fvg.it; 3 Scuola Microbioma Torino, 10100 Torino, Italy; scuolamicrobioma@gmail.com; 4 Department of Medicine, Surgery and Health Sciences, University of Trieste, Strada di Fiume 447, 34149 Trieste, Italy

**Keywords:** next-generation sequencing, microbiome, cytokines, autism spectrum disorder, HMOs

## Abstract

Recent evidence has highlighted the role of the gut–brain axis in the progression of autism spectrum disorder (ASD), with significant changes in the gut microbiome of individuals with this condition. This report investigates the effects of probiotics and human milk oligosaccharide (HMO) supplements on the gut microbiome, inflammatory cytokine profile, and clinical outcomes in an ASD adolescent with chronic gastrointestinal dysfunction and cognitive impairment. Following treatment, we observed a decrease in proinflammatory cytokines’ concentration alongside *Sutterella* relative abundance, a bacterium reported to be linked with gastrointestinal diseases. Also, we reported a notable increase in mood stability. The study aims to evaluate the use of gut microbiome-based therapy in selected ASD patients, highlighting its potential to improve related clinical symptoms.

## 1. Introduction

Autism spectrum disorder (ASD) is a neurological and developmental disorder that affects how people interact with others, communicate, learn, and behave, and it is manifested by impaired social communication and other repetitive behavioral patterns. ASD is caused by both genetic and environmental factors [1]. 

The ASD incidence rate has increased threefold in the last three decades, not only due to the advanced diagnostic methods but also because of an increase in risk factors [2]. The management costs associated with ASD are high [3] and usually extend throughout an individual’s lifetime.

Communication between the gut and the brain has been shown to affect many neurological conditions including ASD [4]. In the last few years, the research on the microbiome has gained significant importance thanks to the huge improvements in omics technologies [5]. Today, there is accumulated evidence about alterations in the gut microbiome in individuals with ASD [6,7]. For instance, research has shown elevated levels of *Sutterella* species in both gastrointestinal biopsies and fecal samples of children with this disorder [8]. Interestingly, a recent study revealed that *Bacteroides* may exert harmful effects and be responsible for some autistic behaviors in mice regardless of sex [9].

Although there is no therapy for ASD, studies showed a possibility of treating some autistic behaviors and making symptoms milder. Among the promising methods to alleviate symptoms, manipulating the gut microbiota through microbiota transplant [10,11,12] has been shown as a promising therapy for ASD.

Probiotics are living microorganisms that offer benefits to the host organism when ingested in appropriate quantities. They are thought to confer health benefits by promoting a balanced gut microbiota and supporting various physiological functions. Several studies highlighted the benefits of probiotics in the treatment of ASD. In rodent models of ASD, a probiotics combination containing *Lactobacillus* spp. and *Bifidobacterium* spp. reduced social and behavioral symptoms associated with this disorder [13]. Results of a phase Ib study of a combination containing *Lactobacillus reuteri* showed important improvements in behavioral and social scores for ASD patients [14]. 

Human milk oligosaccharides (HMOs) are the third component of human milk after lactose and lipids. More than 200 different types of HMOs have been identified so far, all derived from lactose elongated with other monosaccharides such as galactose, N-acetylglucosamine, N-acetylgalactosamine, fucose, and sialic acid [15]. Humans cannot digest HMOs, with the exception of sialylated HMOs cleaved by intestinal neuraminidase [16], and they are absorbed in only a small amount [17]. On the contrary, different microbial taxa belonging to the gut microbiota are well equipped with a broad range of enzymes for HMOs’ digestion: *Bifidobacteria*, and in particular *Bifidobacterium bifidum*, *Bifidobacterium breve*, and *Bifidobacterium longum* strains, among the first colonizers of the infant’s gut, can secrete glycosides and/or express specific transporters for HMOs’ utilization and degradation [18]. Some members of *Bacteroides* spp. are also able to utilize HMOs, while the growth of a broad range of pathogens, such as some strains of *Enterobacteriaceae*, *Streptococcus* spp., and others, is inhibited by different types of HMOs, as demonstrated both in in vitro and in vivo studies [17,19]. The digestion of HMOs by *Bifidobacteria* and *Bacteroides* fermentation increases the production of short chain fatty acids (SCFAs), especially acetate and butyrate, with local and systemic effects, while HMO-derived metabolites can be used as nutritional substrates by other gut bacteria during cross-feeding microbial interactions [20]. The symbiotic combinations of HMOs and probiotics were reported to exert beneficial effects in several studies [21,22,23]. HMOs support the immune system, fight infections, inhibit pathogens, promote gut health, and support cognitive development [17]. As just described, the establishment of a healthy gut microbiota is linked to HMOs. Indeed, many animal studies show that HMOs can affect brain activity and cognitive development, suggesting that dietary management of the gut microbiota may influence several diseases, including ASD [24].

In the present case report, we investigate the effect of a combined treatment with probiotics and HMO supplements in a young man (17 years old) with ASD, suffering from cognitive impairment and chronic gastrointestinal dysfunction including abdominal pain and chronic constipation. By evaluating the impact on gut microbiome composition, inflammation, and related clinical symptoms, we provide additional insights into the potential use of microbiome-based therapies.

## 2. Materials and Methods

### 2.1. Clinical Samples and Therapy

Four fecal samples were collected at 4 different time points: T_0_ (October 2020) represents the physiological condition of the patient before the treatment, T_1_ corresponds to evaluations made after 6 months of therapy, T_2_ corresponds to the evaluation 1 year after the end of the treatment, while T_3_ is the condition of the ASD individual after 2 years without the treatment. 

The patient received antibiotics, precisely, Rifaximin (2 tablets three times daily for 10 days), which is used to treat conditions related to gut microbiota imbalance, potentially modulating intestinal homeostasis [25]. Antibiotic therapy was then followed by a daily intake of 12.5 billion *Bifidobacterium lactis* Bi-07 and 12.5 billion *Lactobacillus acidophilus* NCFM^®^ (North Carolina Food Microbiology). Alongside the probiotics, supplementary therapy included 2’-FL fucosylated HMO (250 mg), with one capsule recommended daily [26,27]. The regimen also included rice extract tocopherols (1 capsule per day preferably in the morning on an empty stomach and away from antibiotic therapy) [28], monounsaturated fatty acids (oleic acid), and polyunsaturated linoleic acids (1 capsule of 250 mg twice daily), known for their antioxidant, anti-inflammatory, hypocholesterolemic [29,30], and hypotriglyceridemic properties. Additionally, sunflower oil (1 tablespoon per day), rich in vitamin E with antioxidant properties, was included, which was reported to promote intestinal health [31].

The study was conducted in accordance with the Declaration of Helsinki and approved by the Institutional Review Board of Institute for Maternal and Child Health IRCCS Burlo Garofolo (protocol code 37902 dd. 12.10.2021).

Written informed consent has been obtained from the patient for all procedures and to publish this paper.

### 2.2. Biological Sampling Procedures, DNA Extraction, and NGS Sequencing 

Upon arrival at the laboratory, the fecal samples were immediately stored at −80 °C until the moment of analysis. Total nucleic acids were extracted using the Maxwell Promega extractor with a CSC DNA Blood Kit (Promega, Madison, WI, USA), following the manufacturer’s instructions. Briefly, 100–200 mg of stool was lysed, and DNA was eluted in a final volume of 50 µL.

The bacterial composition profiling was achieved by sequencing the V3 region of the 16S rRNA gene following the library preparation procedure previously described [32]. Template preparation was carried out by the Ion OneTouch™ 2 System (Life Technologies, Gran Island, New York, NY, USA), with the Ion PGM Hi-Q View OT2 kit (Life Technologies, New York, NY, USA), and for quality control, the Qubit^®^ 2.0 Fluorometer was used. Stool samples were sequenced with the Ion PGM™ System technology by using the Ion PGM Hi-Q View sequencing kit (Life Technologies, New York, NY, USA). For the raw data processing, the QIIME 2.0 software, version 2022.2, was used. Reads with Q ≥ 20 and a read length of 180 bp, after DADA2 denoising, were retained for the analysis. The taxonomy assignment was performed by aligning the read to Silva v138 database, with a BLAST+ consensus. Statistical analysis was performed using R (version 4.4.0).

### 2.3. Dosage of Immune Factors

The concentration (pg/mL) of 27 cytokines, chemokines, and growth factors was dosed in all 4 stool samples, using magnetic bead-based multiplex immunoassays (Bioplex ProTM human cytokine 27-plex panel, Bio-Rad Laboratories, Milan, Italy), according to the pre-optimized protocol [32]. Cytokines tested include FGF basic, Eotaxin, G-CSF, GM-CSF, IFN-γ, IL-1β, IL-1ra, IL-2, IL-4, IL-5, IL-6, IL-7, IL-8, IL-9, IL-10, IL-12 (p70), IL-13, IL-15, IL-17, IP-10, MCP-1 (MCAF), MIP-1α, MIP-1β, PDGF-BB, RANTES, TNF-α, and VEGF. The concentrations of the cytokines were acquired using the Bio-Plex-200 system (Bio-Rad Corp., Hercules, CA, USA) and Bio-Plex Manager software (v.6, Bio-Rad). The Kruskal–Wallis test was performed to test the difference of the immune factors’ concentration over time.

## 3. Results

### 3.1. Microbiota Characterization

Sequencing was performed on four stool samples from a young man diagnosed with ASD, collected at different time points. Microbial composition was analyzed to observe changes in the patient’s intestinal microbiome over time, both during and after the administration of the probiotic/HMO treatment.

**Figure 1:**
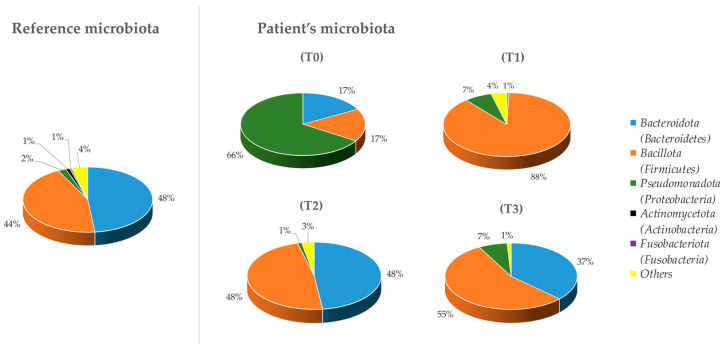
Composition, at the phylum level (Ion PGM™ System technology), of the fecal microbiota in the ASD individual at 4 different time points, compared to a reference microbiota.

Bacterial phyla detected by the analysis were studied and compared (Figure 1). The microbiome changed considerably, especially when comparing T_0_ with the other time points and with the reference microbiome, deduced from the scientific literature [33,34]. Figure 1 shows a substantial difference among the relative abundances of the different phyla: at T_0_, the ASD individual showed, compared to the reference sample, a high abundance of *Pseudomonadota* (66%), which decreased considerably at T_1_ (7%). Furthermore, at T_0_, the *Bacillota* percentage was not high (17%), but this is seen to increase after the treatment, reaching 88% at T_1_ and remaining around 50% at the last two analysis times.

**Figure 2:**
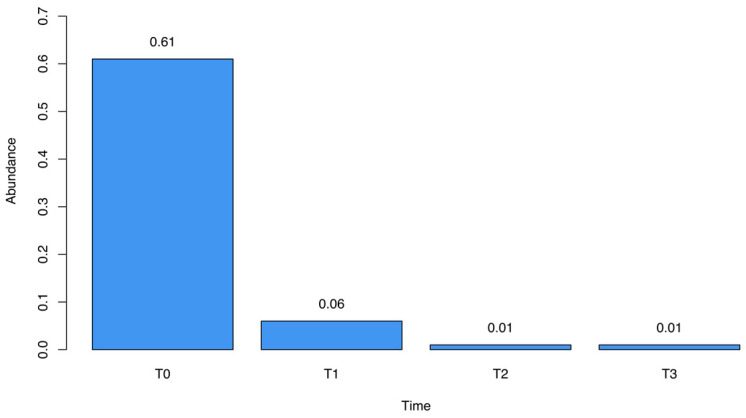
Relative abundance of *Sutterella* spp. over the four different time points described.

We observed marked fluctuations in the abundance of specific bacterial genera throughout the study period. *Sutterella* spp., initially prominent at T_0_ (61%), demonstrated a drastic reduction following treatment, declining to 6% and 1% at subsequent time points (Figure 2). The analysis of the sequencing data highlighted *Sutterella massiliensis* as the predominant species in all the fecal samples.

**Figure 3:**
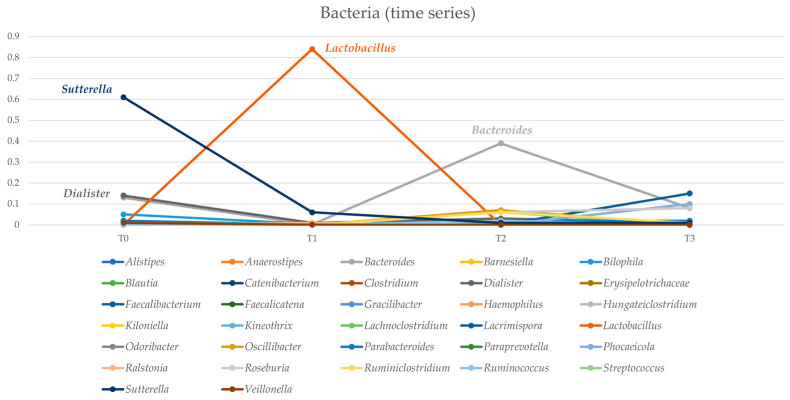
Trend over different times of the relative abundances of bacterial genera detected by the sequencing analysis.

Similarly, *Dialister* spp. exhibited a reduction from 14% at T_0_ to 1% at T_3_. In contrast, *Lactobacillus* spp. showed a substantial increase from T_0_ to T_1_, peaking at 84% in the latter period, before gradually declining. *Bacteroides* abundance fluctuated across analyses, ranging from 13% at T_0_ to 39% at T_2_ and concluding at 8% at T_3_ (Figure 3).

### 3.2. Soluble Immune Mediators

**Figure 4:**
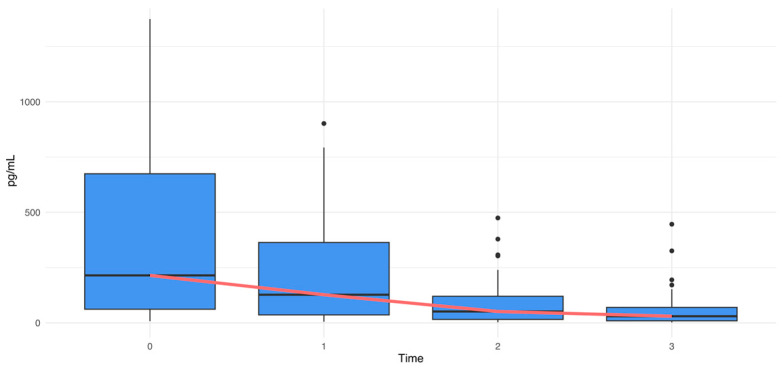
Cytokines’ variation over time (Kruskal–Wallis test|*p*-value < 10^−4^, chi-squared = 21.56). The median trend line is shown in red, and the outlier values are represented as black dots.

The dosage of the concentration (pg/mL) of 27 cytokines, chemokines, and growth factors showed a decline from T_0_ to T_3_, suggesting a potential modulation of immune function over time (Figure 4). All cytokines, both pro-inflammatory and anti-inflammatory ones, decreased over time. The reduction was confirmed by the Kruskal–Wallis test (*p*-value < 10^−4^, chi-squared = 21.56).

**Figure 5:**
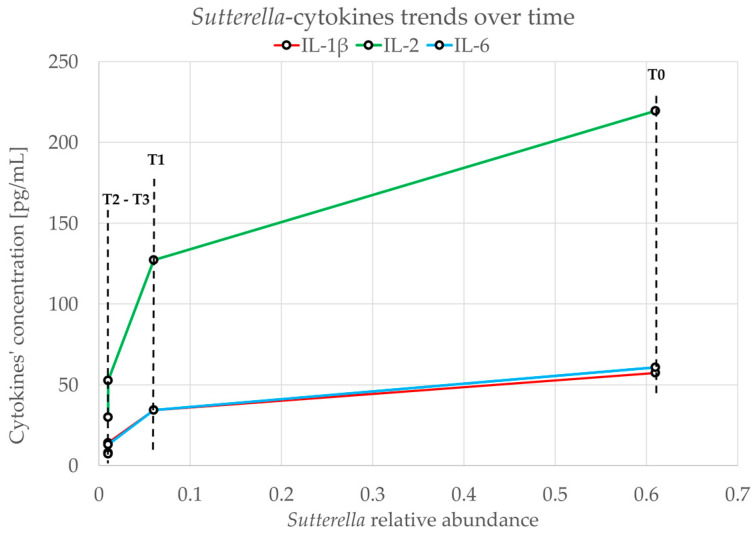
Reduction trend of *Sutterella* and IL-1β, IL-2, and IL-6 over time. The relative abundance is represented on the x-axis, while on the y-axis, the concentration in pg/mL of the three cytokines is taken into consideration.

A direct relationship between each cytokine and *Sutterella* was observed. For example, Figure 5 describes the relationship between IL-1β, IL-2, IL-6, and *Sutterella*. The drop from T_0_ to T_1_ has a reduced slope compared to that which can be observed in the comparison between T_1_–T_2_ and T_2_–T_3_ (Figure 5).

### 3.3. Clinical and Physical Aspects

As the levels of *Sutterella* spp. decreased alongside the reduction in inflammatory factors, the boy achieved a complete recovery from all the symptoms of constipation. Furthermore, this positive shift in his health profile was related to a notable decrease in episodes of aggression and an increase in mood stability. In addition, he developed good behavioral social relations as confirmed by the parents. The improvement in aggressiveness and increased mood stability has been firmly reported by the parents of the young man. Unfortunately, the Aberrant Behavior Checklist (ABC) and Child Behavior Checklist (CBCL) matching the time points of the present case report were not available. 

## 4. Discussion

The use of probiotics in intestinal dysfunction and related inflammation has been documented, and their tolerability and beneficial effect were also reported. For instance, a probiotic blend containing *L. acidophilus* NCFM^®^ and *B. lactis* Bi-07 was used to treat the functional bowel diseases [35]. This clinical study reported by Ringel et al. [35], indeed, highlighted the safety of adopting microbiome-based therapies to improve the clinical condition of subjects characterized by gastrointestinal disorders. 

In another study published in 2013 by Hsiao et al. [36], the gut–microbiome–brain connection was investigated. The results demonstrated that the restoration of gastrointestinal function through microbiota-based treatment led to improvements in communicative, stereotypic, anxiety-like, and sensorimotor behaviors in a mouse model of ASD. A further recent study demonstrated comparable outcomes following treatment with *B. lactis* in a mouse model [37].

Providing additional evidence, research has revealed that a probiotic formulation composed of *Lactobacillus paracasei* HII01, *B. breve*, and *B. longum* exhibited notable improvements in intestinal permeability, lipid profile, obesity index, and metabolic biomarkers in an elderly population [38].

ASD is associated with high levels of inflammation not only in the nerves but also in intestinal tissue. Hence, targeting inflammation pathways could be a potential strategy to alleviate ASD symptoms. Shaaban et al. [39] observed significant improvements in gastrointestinal symptoms in a cohort of autistic children aged 4–9 years following a three-month probiotic treatment regimen composed of *B*. *longum*, *Lactobacillus rhamnosus*, and *L*. *acidophilus*. Similar results were also obtained by other studies [40,41,42,43].

Dysbiosis of the gut microbiota can compromise intestinal barriers. The barrier impairment is reported to be implicated in several diseases, not only in gut-related disorders but also in neurodegenerative conditions like Alzheimer’s and Parkinson’s [44]. Some studies have underscored the effect of the harmful microbiota on brain function in autism and other mental disorders. For example, lipopolysaccharides (LPSs), which are the endotoxins of Gram-negative bacteria, can pass through the damaged intestinal barrier to the brain and induce a proinflammatory environment, potentially leading to pathological consequences [45,46]. Although some studies suggest that intestinal barrier dysfunction is not a consequence of autism [47,48], our results provide strong evidence to consider the link between autism and intestinal permeability.

In our report, we observed a significant decrease in cytokine concentration (Figure 4) in stool samples following HMO-probiotic therapy, an event persisting 2 years after the end of the treatment. This reduction can be explained, at least in part, by the enhanced functionality of the gastrointestinal barrier, due to the restoration of a healthy gut microbiome. An intact epithelial barrier prevents the translocation of pathogens, microbial peptides, and toxins that can trigger inflammatory responses. This restoration can be evidenced, for example, by the reduction in *Sutterella* levels (Figure 2), which was reported to be linked with gastrointestinal diseases for its capacity to degrade IgA [49]. The reduction in *Sutterella* is related to the decrease in the levels of proinflammatory cytokines like IL-1β, IL-2, and IL-6 (Figure 5).

While a previous study [50] did not highlight *Sutterella* as a significant contributor to microbial dysbiosis, our findings suggest that the abundance of *Sutterella* could be a marker for the integrity of the intestinal microbial component. Furthermore, our research indicates alterations in other bacterial genera during treatment, such as *Bacteroides*, which increased to around 37% by the study’s conclusion, nearing its percentage in the reference microbiome (Figure 1). These shifts may have contributed to the favorable outcomes observed following the treatment.

The novelty of the treatment involved the simultaneous intake of *B. lactis* Bi-07 and *L. acidophilus* NCFM^®^, together with 2’-FL fucosylated HMOs. Among benefits related to HMO functions are the development of immune system competence; the protection against infectious disease; the inhibition of pathogens’ epithelial adhesion and biofilm formation; the maturation of the intestinal barrier; the production of mucin, claudin, and occludin proteins; and cognitive development [16,17,51]. In a study conducted by Elison et al. [52], it was reported that HMO supplementation resulted in changes in the gut microbiota composition, with an increase in the relative abundance of *Bifidobacteria* and a reduction in *Firmicutes* and *Proteobacteria*. This effect occurred rapidly and was dose-dependent. No relevant changes in blood parameters were observed, and the supplements were well tolerated at all dosages, with minimal side effects. The results of this study suggest that dietary supplementation with HMOs is an effective method to positively modulate the composition of the intestinal microbiota, promoting the growth of beneficial bacteria, as also reported in our case report. In another study using the Stimulator of the Human Intestinal Microbial Ecosystem (SHIME^®^) (ProDigest-Ghent University, Ghent, Belgium), Šuligoj et al. [26] investigated the effect of HMO-derived metabolites on the gut barrier. An increase in *Bifidobacteria* species and in butyrate production was observed. Moreover, Šuligoj et al. showed a significant increase in the expression of claudin-8 and claudin-5 genes, and a reduction in the secretion of IL-6 for 2′-FL and the mixture 2′-FL/Lacto-N-neotetraose (LNnT) but not for LNnT alone. All together, these data support the beneficial effects of HMOs on the gut barrier and intestinal permeability which is in agreement with our findings.

Overall, our study together with those previously cited provides further support to the hypothesis that the manipulation of the gut microbiota may be beneficial in patients with gastrointestinal disorders and to related specific clinical conditions. Major positive effects on the intestinal barrier can be achieved by the combination of probiotic bacteria and HMOs, also resulting in a general improvement in the subject’s clinical condition.

## 5. Conclusions

In conclusion, we evaluated the effects of a combined treatment with HMOs, *B. lactis* Bi-07, and *L. acidophilus* NCFM^®^ on the gut microbiome composition, inflammatory cytokine profile, and clinical symptoms of a 17-year-old man with ASD. Precisely, we observed a decrease in inflammatory cytokines’ concentration, such as IL-1β, IL-2, and IL-6, together with the reduction in *Sutterella* spp. in stool samples after the combined treatment. The implications of our results are substantial and highlight the need for continued research into microbiome-based therapies, which could pave the way for more effective approaches to the management of gastrointestinal symptoms and related disorders in ASD subjects.

## Data Availability

The raw data supporting the conclusions of this article will be made available by the authors on request.

## References

[1] Chaste P., Leboyer M. (2012). Autism Risk Factors: Genes, Environment, and Gene-Environment Interactions. Dialogues Clin. Neurosci..

[2] Solmi M., Song M., Yon D.K., Lee S.W., Fombonne E., Kim M.S., Park S., Lee M.H., Hwang J., Keller R. (2022). Incidence, Prevalence, and Global Burden of Autism Spectrum Disorder from 1990 to 2019 across 204 Countries. Mol. Psychiatry.

[3] Bieleninik Ł., Gold C. (2021). Estimating Components and Costs of Standard Care for Children with Autism Spectrum Disorder in Europe from a Large International Sample. Brain Sci..

[4] Loh J.S., Mak W.Q., Tan L.K.S., Ng C.X., Chan H.H., Yeow S.H., Foo J.B., Ong Y.S., How C.W., Khaw K.Y. (2024). Microbiota–Gut–Brain Axis and Its Therapeutic Applications in Neurodegenerative Diseases. Signal Transduct. Target. Ther..

[5] Cryan J.F., O’Riordan K.J., Sandhu K., Peterson V., Dinan T.G. (2020). The Gut Microbiome in Neurological Disorders. Lancet Neurol.

[6] Liu S., Li E., Sun Z., Fu D., Duan G., Jiang M., Yu Y., Mei L., Yang P., Tang Y. (2019). Altered Gut Microbiota and Short Chain Fatty Acids in Chinese Children with Autism Spectrum Disorder. Sci. Rep..

[7] Tomova A., Husarova V., Lakatosova S., Bakos J., Vlkova B., Babinska K., Ostatnikova D. (2015). Gastrointestinal Microbiota in Children with Autism in Slovakia. Physiol. Behav..

[8] Wang L., Christophersen C.T., Sorich M.J., Gerber J.P., Angley M.T., Conlon M.A. (2013). Increased Abundance of Sutterella Spp. and Ruminococcus Torques in Feces of Children with Autism Spectrum Disorder. Mol. Autism..

[9] Carmel J., Ghanayem N., Mayouf R., Saleev N., Chaterjee I., Getselter D., Tikhonov E., Turjeman S., Shaalan M., Khateeb S. (2023). Bacteroides Is Increased in an Autism Cohort and Induces Autism-Relevant Behavioral Changes in Mice in a Sex-Dependent Manner. npj Biofilms Microbiomes.

[10] Kang D.-W., Adams J.B., Gregory A.C., Borody T., Chittick L., Fasano A., Khoruts A., Geis E., Maldonado J., McDonough-Means S. (2017). Microbiota Transfer Therapy Alters Gut Ecosystem and Improves Gastrointestinal and Autism Symptoms: An Open-Label Study. Microbiome.

[11] Kang D.-W., Adams J.B., Coleman D.M., Pollard E.L., Maldonado J., McDonough-Means S., Caporaso J.G., Krajmalnik-Brown R. (2019). Long-Term Benefit of Microbiota Transfer Therapy on Autism Symptoms and Gut Microbiota. Sci. Rep..

[12] Wang J., Cao Y., Hou W., Bi D., Yin F., Gao Y., Huang D., Li Y., Cao Z., Yan Y. (2023). Fecal Microbiota Transplantation Improves VPA-Induced ASD Mice by Modulating the Serotonergic and Glutamatergic Synapse Signaling Pathways. Transl. Psychiatry.

[13] Mintál K., Tóth A., Hormay E., Kovács A., László K., Bufa A., Marosvölgyi T., Kocsis B., Varga A., Vizvári Z. (2022). Novel Probiotic Treatment of Autism Spectrum Disorder Associated Social Behavioral Symptoms in Two Rodent Models. Sci. Rep..

[14] Schmitt L.M., Smith E.G., Pedapati E.V., Horn P.S., Will M., Lamy M., Barber L., Trebley J., Meyer K., Heiman M. (2023). Results of a Phase Ib Study of SB-121, an Investigational Probiotic Formulation, a Randomized Controlled Trial in Participants with Autism Spectrum Disorder. Sci. Rep..

[15] Cheng L., Akkerman R., Kong C., Walvoort M.T.C., de Vos P. (2021). More than Sugar in the Milk: Human Milk Oligosaccharides as Essential Bioactive Molecules in Breast Milk and Current Insight in Beneficial Effects. Crit. Rev. Food Sci. Nutr..

[16] Sprenger N., Tytgat H.L.P., Binia A., Austin S., Singhal A. (2022). Biology of Human Milk Oligosaccharides: From Basic Science to Clinical Evidence. J. Hum. Nutr. Diet..

[17] Rousseaux A., Brosseau C., Le Gall S., Piloquet H., Barbarot S., Bodinier M. (2021). Human Milk Oligosaccharides: Their Effects on the Host and Their Potential as Therapeutic Agents. Front. Immunol..

[18] Kitaoka M. (2012). Bifidobacterial Enzymes Involved in the Metabolism of Human Milk Oligosaccharides123. Adv. Nutr..

[19] Salli K., Hirvonen J., Siitonen J., Ahonen I., Anglenius H., Maukonen J. (2021). Selective Utilization of the Human Milk Oligosaccharides 2′-Fucosyllactose, 3-Fucosyllactose, and Difucosyllactose by Various Probiotic and Pathogenic Bacteria. J. Agric. Food Chem..

[20] Kiely L.J., Busca K., Lane J.A., van Sinderen D., Hickey R.M. (2023). Molecular Strategies for the Utilisation of Human Milk Oligosaccharides by Infant Gut-Associated Bacteria. FEMS Microbiol. Rev..

[21] Nolan L.S., Rimer J.M., Good M. (2020). The Role of Human Milk Oligosaccharides and Probiotics on the Neonatal Microbiome and Risk of Necrotizing Enterocolitis: A Narrative Review. Nutrients.

[22] Huang X., Liu R., Wang J., Bao Y., Yi H., Wang X., Lu Y. (2024). Preparation and Synbiotic Interaction Mechanism of Microcapsules of Bifidobacterium Animalis F1-7 and Human Milk Oligosaccharides (HMO). Int. J. Biol. Macromol..

[23] Button J.E., Cosetta C.M., Reens A.L., Brooker S.L., Rowan-Nash A.D., Lavin R.C., Saur R., Zheng S., Autran C.A., Lee M.L. (2023). Precision Modulation of Dysbiotic Adult Microbiomes with a Human-Milk-Derived Synbiotic Reshapes Gut Microbial Composition and Metabolites. Cell Host Microbe.

[24] Vázquez E., Barranco A., Ramírez M., Gruart A., Delgado-García J.M., Martínez-Lara E., Blanco S., Martín M.J., Castanys E., Buck R. (2015). Effects of a Human Milk Oligosaccharide, 2′-Fucosyllactose, on Hippocampal Long-Term Potentiation and Learning Capabilities in Rodents. J. Nutr. Biochem..

[25] Lopetuso L.R., Petito V., Scaldaferri F., Gasbarrini A. (2015). Gut Microbiota Modulation and Mucosal Immunity: Focus on Rifaximin. Mini. Rev. Med. Chem..

[26] Šuligoj T., Vigsnæs L.K., Abbeele P.V.d., Apostolou A., Karalis K., Savva G.M., McConnell B., Juge N. (2020). Effects of Human Milk Oligosaccharides on the Adult Gut Microbiota and Barrier Function. Nutrients.

[27] Vandenplas Y., Berger B., Carnielli V.P., Ksiazyk J., Lagström H., Sanchez Luna M., Migacheva N., Mosselmans J.-M., Picaud J.-C., Possner M. (2018). Human Milk Oligosaccharides: 2′-Fucosyllactose (2′-FL) and Lacto-N-Neotetraose (LNnT) in Infant Formula. Nutrients.

[28] Colombo R., Moretto G., Barberis M., Frosi I., Papetti A. (2023). Rice Byproduct Compounds: From Green Extraction to Antioxidant Properties. Antioxidants.

[29] Chen J., Liu H. (2020). Nutritional Indices for Assessing Fatty Acids: A Mini-Review. Int. J. Mol. Sci..

[30] Wu H., Xu L., Ballantyne C.M. (2020). Dietary and Pharmacological Fatty Acids and Cardiovascular Health. J. Clin. Endocrinol. Metab..

[31] Nederveen J.P., Mastrolonardo A.J., Xhuti D., Di Carlo A., Manta K., Fuda M.R., Tarnopolsky M.A. (2023). Novel Multi-Ingredient Supplement Facilitates Weight Loss and Improves Body Composition in Overweight and Obese Individuals: A Randomized, Double-Blind, Placebo-Controlled Clinical Trial. Nutrients.

[32] Campisciano G., Zanotta N., Quadrifoglio M., Careri A., Torresani A., Cason C., De Seta F., Ricci G., Comar M., Stampalija T. (2023). The Bacterial DNA Profiling of Chorionic Villi and Amniotic Fluids Reveals Overlaps with Maternal Oral, Vaginal, and Gut Microbiomes. Int. J. Mol. Sci..

[33] Salguero M.V., Al-Obaide M.A.I., Singh R., Siepmann T., Vasylyeva T.L. (2019). Dysbiosis of Gram-Negative Gut Microbiota and the Associated Serum Lipopolysaccharide Exacerbates Inflammation in Type 2 Diabetic Patients with Chronic Kidney Disease. Exp. Ther. Med..

[34] Hylemon P.B., Harris S.C., Ridlon J.M. (2018). Metabolism of Hydrogen Gases and Bile Acids in the Gut Microbiome. FEBS Lett..

[35] Ringel-Kulka T., Palsson O.S., Maier D., Carroll I., Galanko J.A., Leyer G., Ringel Y. (2011). Probiotic Bacteria Lactobacillus Acidophilus NCFM and Bifidobacterium Lactis Bi-07 versus Placebo for the Symptoms of Bloating in Patients with Functional Bowel Disorders: A Double-Blind Study. J. Clin. Gastroenterol..

[36] Hsiao E.Y., McBride S.W., Hsien S., Sharon G., Hyde E.R., McCue T., Codelli J.A., Chow J., Reisman S.E., Petrosino J.F. (2013). The Microbiota Modulates Gut Physiology and Behavioral Abnormalities Associated with Autism. Cell.

[37] Miao Z., Chen L., Zhang Y., Zhang J., Zhang H. (2024). Bifidobacterium Animalis Subsp. Lactis Probio-M8 Alleviates Abnormal Behavior and Regulates Gut Microbiota in a Mouse Model Suffering from Autism. mSystems.

[38] Chaiyasut C., Sivamaruthi B.S., Lailerd N., Sirilun S., Khongtan S., Fukngoen P., Peerajan S., Saelee M., Chaiyasut K., Kesika P. (2022). Probiotics Supplementation Improves Intestinal Permeability, Obesity Index and Metabolic Biomarkers in Elderly Thai Subjects: A Randomized Controlled Trial. Foods.

[39] Shaaban S.Y., El Gendy Y.G., Mehanna N.S., El-Senousy W.M., El-Feki H.S.A., Saad K., El-Asheer O.M. (2018). The Role of Probiotics in Children with Autism Spectrum Disorder: A Prospective, Open-Label Study. Nutr. Neurosci..

[40] Santocchi E., Guiducci L., Prosperi M., Calderoni S., Gaggini M., Apicella F., Tancredi R., Billeci L., Mastromarino P., Grossi E. (2020). Effects of Probiotic Supplementation on Gastrointestinal, Sensory and Core Symptoms in Autism Spectrum Disorders: A Randomized Controlled Trial. Front. Psychiatry.

[41] Mensi M.M., Rogantini C., Marchesi M., Borgatti R., Chiappedi M. (2021). Lactobacillus Plantarum PS128 and Other Probiotics in Children and Adolescents with Autism Spectrum Disorder: A Real-World Experience. Nutrients.

[42] Meguid N.A., Mawgoud Y.I.A., Bjørklund G., Mehanne N.S., Anwar M., Effat B.A.E.-K., Chirumbolo S., Elrahman M.M.A. (2022). Molecular Characterization of Probiotics and Their Influence on Children with Autism Spectrum Disorder. Mol. Neurobiol..

[43] Niu M., Li Q., Zhang J., Wen F., Dang W., Duan G., Li H., Ruan W., Yang P., Guan C. (2019). Characterization of Intestinal Microbiota and Probiotics Treatment in Children With Autism Spectrum Disorders in China. Front. Neurol..

[44] Denman C.R., Park S.M., Jo J. (2023). Gut-Brain Axis: Gut Dysbiosis and Psychiatric Disorders in Alzheimer’s and Parkinson’s Disease. Front. Neurosci..

[45] Srikantha P., Mohajeri M.H. (2019). The Possible Role of the Microbiota-Gut-Brain-Axis in Autism Spectrum Disorder. Int. J. Mol. Sci..

[46] Kim H.S., Kim S., Shin S.J., Park Y.H., Nam Y., Kim C.W., Lee K.W., Kim S.-M., Jung I.D., Yang H.D. (2021). Gram-Negative Bacteria and Their Lipopolysaccharides in Alzheimer’s Disease: Pathologic Roles and Therapeutic Implications. Transl. Neurodegener..

[47] Fond G., Boukouaci W., Chevalier G., Regnault A., Eberl G., Hamdani N., Dickerson F., Macgregor A., Boyer L., Dargel A. (2015). The “Psychomicrobiotic”: Targeting Microbiota in Major Psychiatric Disorders: A Systematic Review. Pathol. Biol..

[48] Horvath K., Papadimitriou J.C., Rabsztyn A., Drachenberg C., Tildon J.T. (1999). Gastrointestinal Abnormalities in Children with Autistic Disorder. J. Pediatr..

[49] Kaakoush N.O. (2020). Sutterella Species, IgA-Degrading Bacteria in Ulcerative Colitis. Trends Microbiol..

[50] Hiippala K., Kainulainen V., Kalliomäki M., Arkkila P., Satokari R. (2016). Mucosal Prevalence and Interactions with the Epithelium Indicate Commensalism of *Sutterella* spp.. Front. Microbiol..

[51] Bhowmik A., Chunhavacharatorn P., Bhargav S., Malhotra A., Sendrayakannan A., Kharkar P.S., Nirmal N.P., Chauhan A. (2022). Human Milk Oligosaccharides as Potential Antibiofilm Agents: A Review. Nutrients.

[52] Elison E., Vigsnaes L.K., Rindom Krogsgaard L., Rasmussen J., Sørensen N., McConnell B., Hennet T., Sommer M.O.A., Bytzer P. (2016). Oral Supplementation of Healthy Adults with 2′-O-Fucosyllactose and Lacto-N-Neotetraose Is Well Tolerated and Shifts the Intestinal Microbiota. Br. J. Nutr..

